# The impact of climate change on the distribution of two threatened Dipterocarp trees

**DOI:** 10.1002/ece3.2846

**Published:** 2017-03-05

**Authors:** Jiban C. Deb, Stuart Phinn, Nathalie Butt, Clive A. McAlpine

**Affiliations:** ^1^ School of Geography, Planning and Environmental ManagementRemote Sensing Research CentreThe University of QueenslandBrisbaneQldAustralia; ^2^ Department of Forestry and Environmental ScienceSchool of Agriculture and Mineral SciencesShahjalal University of Science and TechnologySylhetBangladesh; ^3^ School of Biological SciencesARC Centre of Excellence for Environmental DecisionsThe University of QueenslandBrisbaneQldAustralia; ^4^ School of Geography, Planning and Environmental ManagementCentre for Biodiversity and Conservation ScienceThe University of QueenslandBrisbaneQldAustralia

**Keywords:** climate change, conservation planning, Dipterocarp trees, forest fragmentation, species distribution

## Abstract

Two ecologically and economically important, and threatened Dipterocarp trees Sal (*Shorea robusta*) and Garjan (*Dipterocarpus turbinatus*) form mono‐specific canopies in dry deciduous, moist deciduous, evergreen, and semievergreen forests across South Asia and continental parts of Southeast Asia. They provide valuable timber and play an important role in the economy of many Asian countries. However, both Dipterocarp trees are threatened by continuing forest clearing, habitat alteration, and global climate change. While climatic regimes in the Asian tropics are changing, research on climate change‐driven shifts in the distribution of tropical Asian trees is limited. We applied a bioclimatic modeling approach to these two Dipterocarp trees Sal and Garjan. We used presence‐only records for the tree species, five bioclimatic variables, and selected two climatic scenarios (RCP4.5: an optimistic scenario and RCP8.5: a pessimistic scenario) and three global climate models (GCMs) to encompass the full range of variation in the models. We modeled climate space suitability for both species, projected to 2070, using a climate envelope modeling tool “MaxEnt” (the maximum entropy algorithm). Annual precipitation was the key bioclimatic variable in all GCMs for explaining the current and future distributions of Sal and Garjan (Sal: 49.97 ± 1.33; Garjan: 37.63 ± 1.19). Our models predict that suitable climate space for Sal will decline by 24% and 34% (the mean of the three GCMs) by 2070 under RCP4.5 and RCP8.5, respectively. In contrast, the consequences of imminent climate change appear less severe for Garjan, with a decline of 17% and 27% under RCP4.5 and RCP8.5, respectively. The findings of this study can be used to set conservation guidelines for Sal and Garjan by identifying vulnerable habitats in the region. In addition, the natural habitats of Sal and Garjan can be categorized as low to high risk under changing climates where artificial regeneration should be undertaken for forest restoration.

## 1 Introduction

Global climate change has produced numerous shifts in the distribution of species over the last three decades and will act as a major cause of species extinction in the near future, either directly or synergistically with other extinction drivers (Akçakaya, Butchart, Watson, & Pearson, 2014; Pacifici et al., 2015; Pearson et al., 2014; Thomas et al., 2004). The potential for large increases in global mean temperatures (e.g., 4.3 ± 0.7°C) by 2100 has significant implications for species and forest ecosystems (Butt, Pollock, & McAlpine, 2013; Pacifici et al., 2015). In the context of understanding ecological responses to climate change, regional changes that are highly spatially heterogeneous may be more relevant than approximated global averages (Walther et al., 2002). Among the four global climate domains (tropical, subtropical, temperate, and boreal), the tropical biome has the highest rate of forest destruction and degradation (Achard et al., 2002; Hansen et al., 2013; Laurance, 2004; Morris, 2010). Therefore, forest–climate interactions in highly modified tropical landscapes are becoming one of the most important subjects of research in conservation ecology (e.g., Laurance, 2004; Wiegand, Revilla, & Moloney, 2005; Wilson et al., 2016).

The climate of South and northern continental Southeast Asia is monsoonal with a large‐scale seasonal reversal of the wind regime and summer‐dominant rainfall (Loo, Billa, & Singh, 2015). In this region, climate change is driving increasing air temperatures and changes in rainfall regimes (Loo et al., 2015; Sivakumar & Stefanski, 2011). Climate change projections suggest a significant acceleration of warming, increasing annual rainfall, and increases in extreme climate events such as floods, drought, and cyclones by 2100 (IPCC, 2013; Loo et al., 2015). The predicted increase in temperature by the late 21st century and early 22nd century will cause frequent changes and shifts in monsoon precipitation of up to 70% below normal levels (Schewe & Levermann, 2012), and monsoons may be delayed by up to 15 days (Schewe & Levermann, 2012). Small‐scale regional circulations are more vulnerable to monsoonal variations, and therefore, temporal and spatial distributions of monsoonal rainfall cannot be represented by general measurements (Loo et al., 2015). The increasing intensity of rainfall during the monsoon season is the major source of extreme climate events such as floods and landslides, which have the potential to affect vegetation (Loo et al., 2015). In some regions, droughts associated with significant changes in tree physiological characteristics (e.g., plant‐extractable water capacity of soil; annual evapotranspiration rate, etc.) could result in regional die‐offs in some species (e.g., Breshears et al., 2005). However, the impacts of climate change on tree species widely distributed over many countries, ecoregions (large units of land containing a geographically distinct assemblage of natural communities and environmental conditions), and topographies (Corlett & Lafrankie, 1998) in Asia have not been widely investigated (e.g., Pacifici et al., 2015; Thomas et al., 2004).

Among the biotic components of forests, trees are one of the earliest groups to be affected by climate change, through changes in phenology and distribution, and these changes could have cascading effects on the functioning of forest ecosystems (Butt et al., 2015; Cleland, Chuine, Menzel, Mooney, & Schwartz, 2007; Corlett & Lafrankie, 1998). Although trees generally respond slowly to climate change, their long life spans suggest they will be unlikely to adapt fast enough to avoid negative impacts of climate change, such as heat and moisture stress and resulting high mortality rates (Margrove et al., 2015; Solomon & Kirilenko, 1997). The indirect effect of changes in tree flowering and fruiting phenology on pollinators and seed dispersal agents (e.g., mammals, birds, and insects) that rely on periodically available plant resources for their survival, may be more serious than the direct effects (Butt et al., 2015; Corlett & Lafrankie, 1998).

The family Dipterocarpaceae comprises approximately 510 species and 16 genera, with 13 genera and 470 species largely restricted to South and Southeast Asia (Appanah & Turnbull, 1998). Dipterocarp forests play an important role in the economy of many South and Southeast Asian countries and dominate the international tropical timber market (Appanah & Turnbull, 1998; Poore, 1989). Dipterocarps are highly variable in terms of flowering and fruiting phenology, ecological characteristics, and geographical ranges, as they occur in evergreen, semievergreen, and deciduous forests (Appanah & Turnbull, 1998). Climatic or geographical variations, along with increasing habitat destruction, are considered key threats for Asian Dipterocarp forests. Among the 13 genera in South and Southeast Asia, the *Shorea* and *Dipterocarpus* are the first and third most diverse genera, respectively, and are important components of Dipterocarp forest ecosystems (Soepadmo, Guan, & Kong, 2004). While most of the species of these two genera are currently listed as threatened in different categories (i.e., 109 and 34 critically endangered species for *Shorea* and *Dipterocarpus* respectively), and at least one species from each genus is now regionally extinct (*Shorea cuspidata* in Malaysia and *Dipterocarpus cinereus* in Indonesia), their status is due to be reviewed (IUCN Species Survival Commission, 2015). The dominant Dipterocarp trees Sal (*Shorea robusta*) and Garjan (*Dipterocarpus turbinatus*) of South and northern continental Southeast Asia form mono‐specific canopies in dry deciduous, moist deciduous, evergreen, and semievergreen forests (Appanah & Turnbull, 1998; Gautam & Devoe, 2006). Further, Sal and Garjan forest ecosystems are the natural habitat of many threatened animal species (e.g., *Elephas maximus*,* Ursus thibetanus*). Projected climate change impacts on Sal and Garjan species have the potential to trigger significant ecosystem‐level responses.

Sal is a timber‐yielding dominant tree that occurs commonly on the plains and lower foothills of the Himalayas and is distributed both in the tropical moist and in the dry deciduous forests of India, Bangladesh, Nepal, and Bhutan (Gautam & Devoe, 2006). Sal forests naturally occur in ecoregions with a mean annual temperature ranging from 22 to 27°C and mean annual rainfall of 1,000 to 2,000 mm (Das & Alam, 2001; Gautam & Devoe, 2006). Although Sal is listed as a “least concern” species in the IUCN Red list (IUCN Species Survival Commission, 2015), recurrent anthropogenic disturbances such as overexploitation, deforestation, and encroachment combined with climate change, are major threats to Sal forests (Kushwaha & Nandy, 2012). Results from previous work suggest that the natural distribution of Sal has contracted very rapidly over the last few decades, and it is thus highly vulnerable to climate change (Chitale & Behera, 2012; Deb, Salman, Halim, Chowdhury, & Roy, 2014; Sarker, Deb, & Halim, 2011). Garjan is a “critically endangered” (IUCN Species Survival Commission, 2015) commercially important Dipterocarp tree naturally distributed in the tropical evergreen, semievergreen, and deciduous forests of Bangladesh, India, Myanmar, Thailand, Cambodia, and Vietnam (Ashton, 1998). Garjan forests are located in wide bioclimatic regions characterized by temperature range of 15.6–40.6°C and annual rainfall of 1,520 to 5,080 mm (Das & Alam, 2001). Garjan timber is used for lorry bodies, boat building, railway sleepers, transmission poles, and other construction purposes (Das & Alam, 2001). It is potentially vulnerable to anthropogenic climate change due to the interaction with existing anthropogenic pressures such as overextraction, deforestation, and forest degradation (Ashton, 1998).

Several Asian countries, including Thailand, Philippines, China, Sri Lanka, Vietnam, and Bangladesh, have imposed logging bans to halt deforestation and conserve forest resources (Sarker et al., 2011). However, the trend of deforestation and associated biodiversity loss has called into question the effectiveness of these bans, and factors such as lack of effective conservation policies and accounting for climate risks also hinder the success of forest conservation and restoration (Sarker et al., 2011). Species distribution models (SDMs) are useful for documenting biodiversity and understanding the effects of climate‐ and human‐induced changes (Dale et al., 2001; Franklin, 2010; Loiselle et al., 2003; Saatchi, Buermann, Ter Steege, Mori, & Smith, 2008). Consequently, conservation practitioners have been increasingly using habitat suitability models and evaluating the results critically and cautiously to make management decisions (Loiselle et al., 2003; Saatchi et al., 2008).

The aim of this paper was to assess the vulnerability of two Dipterocarp trees (Sal and Garjan) of South and Southeast Asia to climate change by modeling their future distributions under two IPCC Representative Concentration Pathway (RCP) scenarios. We projected the potential distributions for both species in 2070 under two climate scenarios (RCP4.5 and RCP8.5). This will allow the identification of future suitable climate space for these Dipterocarp trees and help inform conservation priorities for these threatened species in the region.

## 2 Methods

### 2.1 Species occurrence data

**Figure 1:**
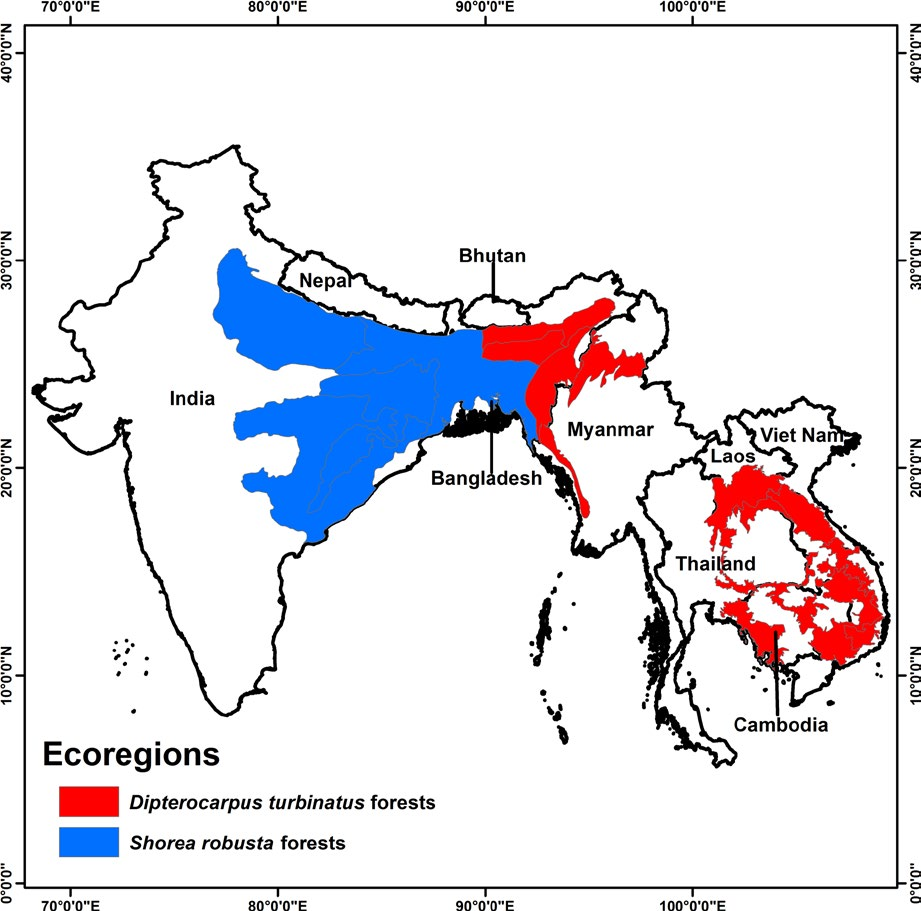
Distribution of *Dipterocarpus turbinatus* and *Shorea robusta* forests (Appanah & Turnbull, 1998; Champion & Seth, 1968; Gautam & Devoe, 2006; Huda et al., 2006) was matched with the ecoregions in South and Southeast Asia (Peel et al., 2007; Table S1 for details). The red polygon depicts the ecoregions for *D. turbinatus* dominant in the evergreen, semievergreen, and deciduous forests of Bangladesh, India, Myanmar, Thailand, Cambodia, and Vietnam, whereas the blue polygon depicts the ecoregions for *S. robusta* dominant in tropical moist and dry deciduous forests of India, Bangladesh, Nepal, and Bhutan

We combined the presence‐only records of Sal and Garjan from a variety of sources including field survey, online database Global Biodiversity Information Facility (http://www.gbif.org/), and literature records. To reduce potential errors in species locations, records were “cleaned,” which included the careful review of literature for each species (Appanah & Turnbull, 1998; Champion & Seth, 1968; Das & Alam, 2001) and the removal of duplicate locations. Finally, we selected 787 and 533 records for Sal and Garjan, respectively, to model their distributions. Sal dominates tropical moist and dry deciduous forests, and Garjan dominates or codominates evergreen, semievergreen, and deciduous forest ecosystems in tropical Asia (Appanah & Turnbull, 1998; Champion & Seth, 1968; Gautam & Devoe, 2006; Huda, Uddin, Haque, Mridha, & Bhuiyan, 2006). We clipped the ecoregions for South and Southeast Asia from the Köppen–Geiger climate classification of the world (Peel, Finlayson, & McMahon, 2007) and combined them with the distributions of the two Dipterocarps to show their ecoregions in tropical Asia (Figure 1 and Table S1).

### 2.2 Bioclimatic variables

We initially considered 19 bioclimatic variables (11 temperature and eight precipitation metrics) from the WorldClim database (Hijmans, Cameron, Parra, Jones, & Jarvis, 2005). All the bioclimatic layers were 1‐km resolution, and we processed all environmental layers using the same extent, cell size, and projection system (WGS84 Longitude‐Latitude projection), in ArcGIS 10.1. We applied Spearman's rank correlation to test for collinearity between variables at each level, to allow us to exclude highly autocorrelated variables. For instance, if a pair of variable has a correlation coefficient >.7, then they were considered proxies of one another, and one of the variables was removed from the analysis (Table S2). Test model runs identified five of the 19 variables as most correlated with the current distributions: annual mean temperature (BIO1); mean diurnal range (BIO2); temperature seasonality (BIO4); annual precipitation (BIO12); and precipitation seasonality (BIO15).

### 2.3 Climate scenarios

We selected two IPCC RCP scenarios for our study: RCP4.5, an optimistic scenario where emissions peak around 2040, and RCP8.5, a pessimistic scenario, which reflects high levels of energy demand and greenhouse gas emissions, resulting in 8.5 W/m^2^ radiative forcing by 2100 (Moss et al., 2010). We constructed models using current climatic conditions (average for 1950–2000) and projected to the future (given by WorldClim for the range 2061–2080, hereafter referred to as 2070). We used three global climate models (hereafter referred to as GCM) for future climatic conditions: ACCESS1.0; GFDL‐CM3; and HadGEM2‐ES (hereafter referred to as GCM 1, GCM 2, and GCM 3 respectively) (Hijmans et al., 2005; Table S3 for details). The reason behind choosing three GCMs was to encompass the full range of variation in the models in the multimodel ensemble CMIP5 that was released 2010–2014 (Taylor, Stouffer, & Meehl, 2012).

### 2.4 MaxEnt modeling algorithm

We used a machine learning method “maximum entropy algorithm” for modeling changes in species distribution (Phillips, Anderson, & Schapire, 2006; Phillips, Dudík, & Schapire, 2004). MaxEnt derives the probability distribution of species based on georeferenced occurrence records and environmental variables, and the output is continuous. It has advantages over other SDMs as it requires species presence‐only data, and both continuous and categorical variables can be used in MaxEnt (Baldwin, 2009). Recent studies have demonstrated MaxEnt's ability to accurately predict species distribution in a wide range of ecological and geographical regions (Araujo & Guisan, 2006; Elith et al., 2006; Merow, Smith, & Silander, 2013).

Sampling bias is a well‐known issue in presence‐only distribution models and can have significant impacts on the model results (Elith et al., 2011). We created a bias file layer to limit the background points to the occurrence areas for the species and accounting for the preferential use of the sites in the study region (Phillips et al., 2009). This provides MaxEnt with a background file with the same bias as the presence locations of the species (Fig. S1 for details). As the distributions of both species are patchy and occur in different countries (of different areas), we used state boundaries of the countries to limit the background areas for the species (Fig. S1). In the model, 75% of the species presence data were used as training data, and the remaining 25% were used as testing data in order to test the model's predictive strength. We tested different regularization multiplier values and selected the default (i.e., 1) option as it performed best, that is, gave the best representation of the current distribution of both Sal and Garjan species without overfitting the model (see Merow et al., 2013). The maximum number of background points for sampling was kept at 10,000. However, we also checked that increasing the background points (e.g., 100,000) did not change the model. We executed five replicates for each species using repeated split samples to measure the amount of variability in the model and then averaged the results. Maximum numbers of iterations were set to 1,000 to allow the model to have adequate time for convergence, with 1 × 10^−6^ set as the convergence threshold. We used the default “autofeatures,” which includes all features (i.e., linear, quadratic, product, threshold, and hinge features; Merow et al., 2013). Area under the receiver operating characteristic curve, or AUC values, for training and testing data was calculated for each species. We used the jackknife test to measure variable importance and percent contributions of each variable to estimate the influence of environmental variables on each species. As the data were compiled from a variety of sources and likely to have some errors, we used the 10 percentile training presence logistic threshold to define the minimum probability of suitable habitat for the Dipterocarp trees (see Phillips et al., 2006). By using this threshold, we defined suitable habitat to include 90% of the data we used to develop the models (Phillips et al., 2006).

## 3 Results

### 3.1 Predictor variables

Our models predict that the relative contribution of the bioclimatic variables was more or less consistent for all three GCMs (Table 1). The key bioclimatic variable explaining the current and future spatial distributions of Sal and Garjan was annual precipitation (Sal: 49.97 ± 1.33; Garjan: 37.63 ± 1.19). The relative contribution of annual mean temperature to both Sal and Garjan models was almost identical (Sal: 19 ± 1.3; Garjan: 19 ± 1.64). The seasonal climatic variables, that is, temperature seasonality (15.33 ± 0.29) and precipitation seasonality (11.43 ± 0.47), were also important contributors to the Sal models, whereas mean diurnal range (4.2 ± 0.66) was least important. In contrast, temperature seasonality (21.5 ± 0.79) and mean diurnal range (16.53 ± 1.11) were important contributors to the Garjan models, with precipitation seasonality least important (5.23 ± 1.20). The jackknife test results suggest that annual precipitation (BIO12) variable contributed most individually for both models (Fig. S2).

**Table 1:** Summary of the bioclimatic variables used in the MaxEnt models and their percent contribution to each model

Variables	Description	Contribution to MaxEnt models (%)					
		*Shorea robusta*			*Dipterocarpus turbinatus*		
		GCM‐1	GCM‐2	GCM‐3	GCM‐1	GCM‐2	GCM‐3
BIO1	Annual mean temperature	20.3	17.7	19	18.1	21	18.2
BIO2	Mean diurnal range (mean of monthly [max temp–min temp])	3.5	4.3	4.8	15.5	16.4	17.7
BIO4	Temperature seasonality (standard deviation × 100)	15.5	15.5	15	22.1	21.8	20.6
BIO12	Annual precipitation	49.1	51.5	49.3	39	36.8	37.1
BIO15	Precipitation seasonality (coefficient of variation)	11.6	10.9	11.8	5.3	4	6.4

GCM, global climate model.

The AUC values for all three GCM models were better than random (0.5) for both species (mean training AUC of the three GCMs for Sal: 0.897, and for Garjan: 0.825) and showed strong model discrimination ability for predicting changes in species distribution under changing climate scenarios (Table 2). The small differences in the AUC value of training and test cases suggested little overfit in the MaxEnt predictions for both species (Table 2). The AUC standard deviations indicate the overall performance of the models was high, representing a close approximation of the true probability distribution of the Dipterocarp trees (Table 2).

**Table 2:** Results of threshold independent ROC tests for Dipterocarp tree species

Species	Models	Training AUC	Test AUC	AUC standard deviation
*Shorea robusta*	GCM‐1	0.894	0.891	0.012
	GCM‐2	0.897	0.891	0.012
	GCM‐3	0.899	0.886	0.013
*Dipterocarpus turbinatus*	GCM‐1	0.827	0.799	0.025
	GCM‐2	0.823	0.790	0.025
	GCM‐3	0.824	0.794	0.025

AUC values for training (75%) and test (25%) data of the models. The test AUC describes the fit of the model to the test data and gives strong model discrimination ability for predicting changes in species distribution under future climate scenarios.

**Figure 2:**
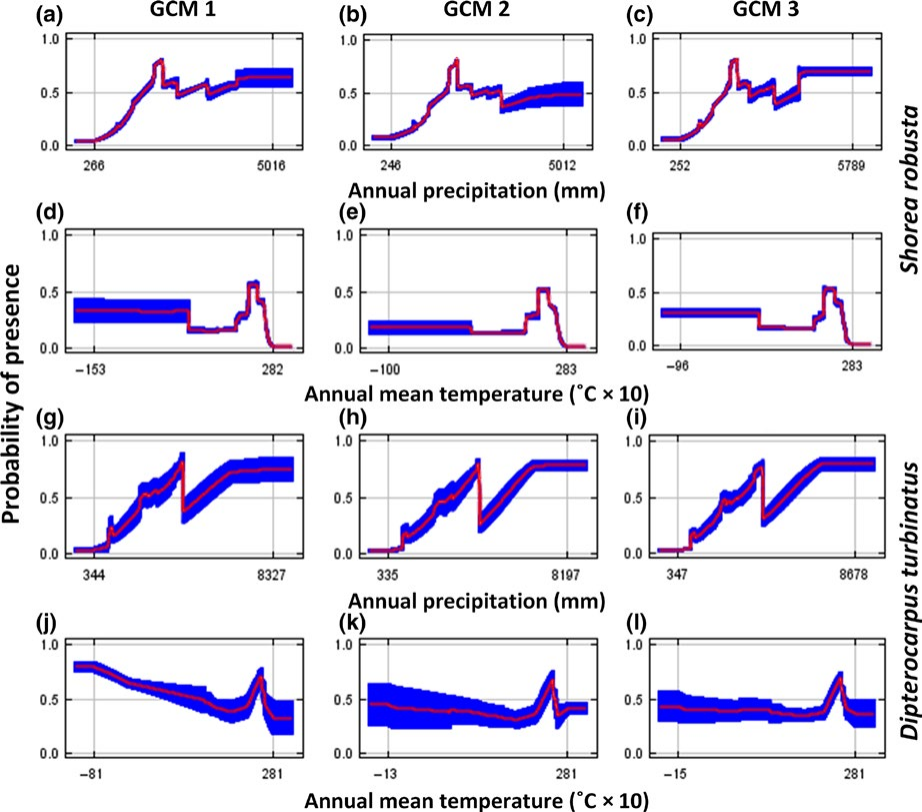
Graphs showing the marginal relationship between each bioclimatic variable and the probability of species occurrence: In the figures, the curves (red) and the mean ± standard deviation (blue) show the response of *Shorea robusta* and *Dipterocarpus turbinatus* to the two most important variables (i.e., keeping all other bioclimatic variables at their average sample value) annual precipitation, and annual mean temperature. The *y*‐axes indicate logistic output (probability of presence). The results suggest that there was an overall positive nonlinear response observed for annual precipitation for both species. The optimum annual mean temperature for the probability of both Sal and Garjan occurrence was approximately 28°C in all models

The individual response curves (marginal responses obtained by keeping all other bioclimatic variables at their average sample value) of the two key variables (annual precipitation and annual mean temperature) portray the relationships between each bioclimatic variable and probability of species occurrence (Figure 2). In Figure 2a–f curves represent the response of annual precipitation and annual mean temperature for three Sal models, respectively. Curves (g–i) and (j–l) represent the response of annual precipitation and annual mean temperature for three Garjan models, respectively. The results exhibit complex but quadratic relationships between bioclimatic variables and the probability of species occurrence. In general, there was an overall positive nonlinear response observed for annual precipitation for both species (Figure 2). The optimum annual mean temperature for the probability of both Sal and Garjan occurrence was approximately 28°C in all models (Figure 2). However, the curves showed a high probability of presence of the species at low temperatures (especially for Garjan; Figure 2 j–l). This might be due to the occurrence of the species in different forest ecosystems with a large range of temperature and elevation.

### 3.2 Variability in climate niches for Dipterocarp trees

**Figure 3:**
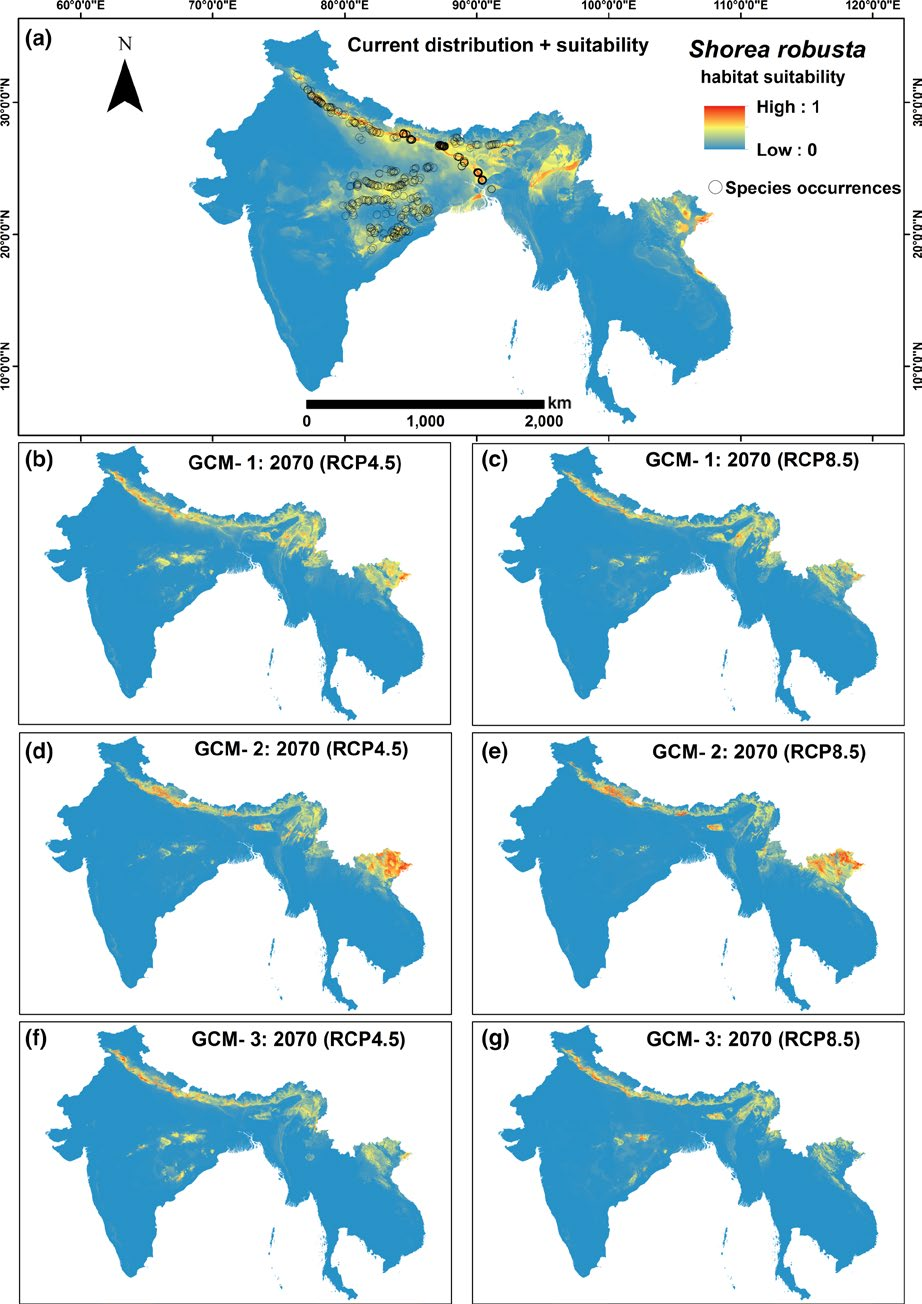
Predicted distribution of *Shorea robusta* species for three global climate models (GCMs): (a) current distribution and suitability; (b, c) scenarios for CGM 1; (d, e) scenarios for GCM 2; and (f, g) scenarios for GCM 3. Modeling results suggest that climatically suitable habitat conditions for Sal will decline by 2070, with an average of 24% and 34% (the mean of three GCMs) under RCP4.5 and RCP8.5, respectively

**Figure 4:**
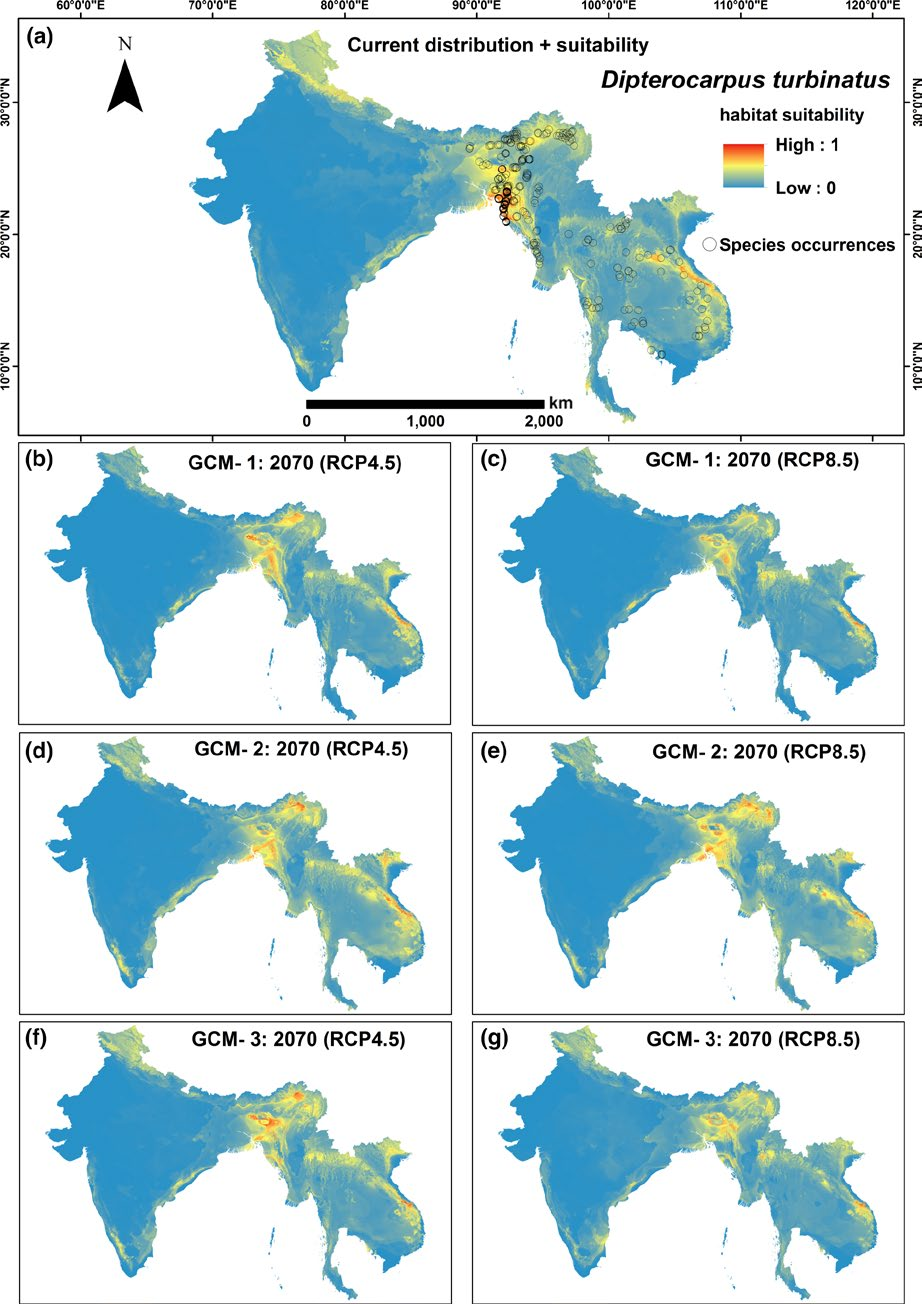
Predicted distribution of *Dipterocarpus turbinatus* species for all global climate models (GCMs): (a) current distribution and suitability; (b, c) scenarios for CGM 1; (d, e) scenarios for GCM 2; and (f, g) scenarios for GCM 3. The consequences of imminent climate change appear less severe for Garjan, with a decline of 17% and 27% (the mean of three GCMs) under RCP4.5 and RCP8.5, respectively

**Figure 5:**
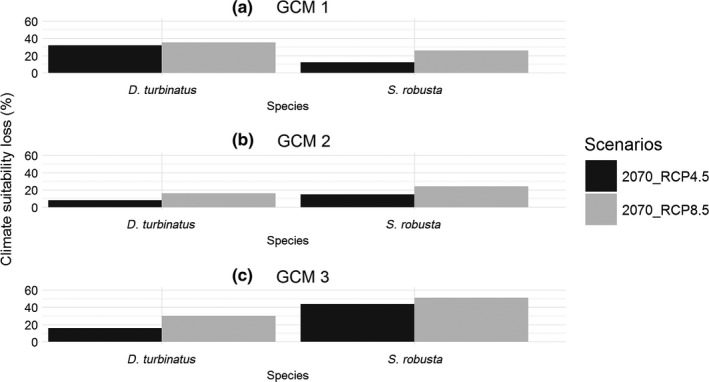
Proportional changes (%) in climate niches for both Dipterocarp species by 2070 under both climate scenarios. Predicted losses of pixel were calculated as a proportion of the pixels occupied in current scenario for the study area. The results of all global climate models suggest that both species are likely to lose climate suitability by 2070 under both climate scenarios

The predicted climatically suitable habitats of Sal and Garjan are shown for all three GCMs in Figures 3 and 4, respectively. The 10th percentile training presence logistic threshold values were used to estimate the suitable and unsuitable climatic niches for both Dipterocarp trees across the study region. The proportional changes in suitable climate niches were derived from the difference between the species' modeled current and future climate niches for each scenario. Our models predicted that suitable climate space for both Sal and Garjan will decline by 2070, under both climate scenarios and for all three GCMs (Figure 5). On average, suitable habitat conditions for Sal will decline by 24% and 34% (the mean of three GCMs) by 2070 under RCP4.5 and RCP8.5, respectively (Figure 5). In contrast, the consequences of climate change appear less severe for Garjan, with a decline of 17% and 27% (the mean of three GCMs) under RCP4.5 and RCP8.5, respectively.

The distribution of Sal in Madhya Pradesh, Chhattisgarh, West Bengal, Odisha, and Jharkhand in India is likely to lose suitable climate space by 2070 (Figure 3). In contrast, the distribution of Sal along the Terai tract in northern India is likely to gain suitable climate space by 2070 (Figure 3b–g). The lower belts of the hilly region, inner Terai, and the protected areas of Nepal, such as Chitwan National Park, Bardia National Park, and Shukla Phat Wildlife Reserve, which support dense Sal forests, are also likely to lose suitable climate niches (Figure 3). The moist deciduous Sal forests in the central and northern region of Bangladesh (e.g., Madhupur National Park and Bhawal National Park) are likely to be affected most by climate change.

The predicted extent of suitable habitat of Garjan is smaller in Bangladesh, Myanmar, Cambodia, Thailand, and Vietnam than in India (Assam, Manipur, Tripura, and Meghalaya). In particular, the Garjan‐dominated semievergreen forests of the Chittagong Hill Tracts region in Bangladesh are likely to face increasing climate stress in the near future, which may lead to local extinctions of this species.

## 4 Discussion

Although the projected distribution scenarios for the three GCMs were not identical in terms of climatically suitable habitat conditions for Sal and Garjan, the relative contribution of all bioclimatic variables used in the models and their AUC values were similar (Table 1 and 2), and the trends of the response curves of the variables for all GCMs were identical (Figure 2). Our results suggest that climate niches for both Dipterocarp trees are likely to come under increasing stress and potentially result in range contraction and distribution shifts across the region during the 21st century.

The study reveals that projected increases in annual precipitation and annual mean temperature may limit the distribution of Sal, as identified by our models (the optimum annual mean temperature was 28°C, and annual precipitation ranges from 1,000 to 2,000 mm; Figure 2 for details; Das & Alam, 2001; Gautam & Devoe, 2006). The variation of temperature seasonality may also regulate the distribution of Sal as it grows in areas where the dry period does not exceed 4 months (Gautam & Devoe, 2006). The predicted shift in the distribution of Sal toward northeast in India is consistent with the findings of a similar study on Sal in India (Chitale & Behera, 2012). Chitale and Behera (2012) predicted the distribution of Sal for the year 2020 under HadClim Emission scenario SRES‐A1B and included all 19 bioclimatic variables without considering their relative contributions. They also found that moisture (annual precipitation) was a key driver of Sal distribution: Our consideration of the relative contribution of the bioclimatic variables revealed that annual mean temperature was also important. Increased rainfall variability and extreme drought conditions in the central and northern parts of Bangladesh may result in unsuitable climate conditions for Sal forests (Shahid, 2010). The projected increase in annual rainfall and variation in temperature seasonality may restrict the distribution of Garjan in the region, with increasing local‐level extinction risk in the Chittagong hill tract regions of Bangladesh (Das & Alam, 2001; Sarker et al., 2011).

Dipterocarp trees are confined to wet climates, with a dry season of 4 months and more abundant in aseasonal than seasonal climates (Ashton, 1988). However, the ecoregions for Sal and Garjan are restricted to monsoon tropics where water availability is seasonally limiting (mean rainfall of driest month < 50 mm; Corlett & Lafrankie, 1998): Significant climatic anomalies such as increasing temperature seasonality and drought conditions may affect the growth of these Dipterocarp trees.

### 4.1 Impacts on Sal and Garjan forest ecosystems

The consequences of climate change may result in the absence of Sal and Garjan either locally or regionally, the disappearance of entire ecosystems, or their replacement by other ecosystem types (Thomas et al., 2004). Changes in precipitation and temperature regimes, including the duration of the dry season, may result in phenological shifts of both Dipterocarp trees, with indirect effects on floral and faunal species dependent on them. Many terrestrial birds, mammals, and insects that rely directly and indirectly on the flowers, fruits, and seeds of Dipterocarps are likely to be adversely affected by climate change (Butt et al., 2015). The continuing deforestation and threats associated with climate change could lead to the extinction of mammal species such as the leopard cat (*Felis bengalensis*), fishing cat (*Felis viverrina*), jungle cat (*Felis chaus*), and small Indian civet (*Viverricula indica*) inhabiting Dipterocarp forests (Alam, Furukawa, Sarker, & Ahmed, 2008; Thomas et al., 2004).

### 4.2 Implications for conservation planning

The findings of our models can be tailored to suit conservation guidelines for Sal and Garjan in South and Southeast Asia by identifying critically vulnerable habitats and potential climatically suitable habitats where artificial regeneration should be undertaken for forest restoration. Our models detected a shift in the distribution of suitable climate space for Sal outside of its natural distribution toward the deciduous and semievergreen forests of northeastern India, Myanmar, Laos, and Vietnam (Figure 3d,e). As a conservation strategy, assisted migration of Sal into these potentially climatically suitable areas may be possible under a wide range of possible future climates (e.g., Hällfors et al., 2016). In addition, the modeling outputs of our study can be used to categorize the natural habitats of Sal and Garjan trees as low to high risk under changing climates in the study region to inform conservation planning. For instance, Sal and Garjan plantations should be preferentially introduced to the climatically suitable sites, and more conservation care for the natural regeneration of these trees should be taken in the sties calculated as high risk under future climates. The rotation period of Sal and Garjan timber may be shortened in those sites and replaced with other species assessed as more suitable under changing climatic conditions.

Forests play an important role in the global carbon cycle as they hold more carbon than the atmosphere (Pan et al., 2011). Sal and Garjan are the long rotation species in South and Southeast Asia and are important for ecosystem functioning and carbon storage. Therefore, small changes in their distributions can have large implications in terms of carbon storage and stocks as they are distributed over a large area in Asia (e.g., Sal forests cover over 11 million ha in India, Bangladesh, and Nepal). Bioclimatic and ecological traits of Dipterocarp species in a particular forest ecosystem are very important for successful forest management, as climate change can drive significant alterations in forest site conditions (Falk & Mellert, 2011). This type of study, of changes in suitable climate space, and therefore the distribution of tree species, could inform forest carbon management.

### 4.3 Future research directions

Although MaxEnt cannot be viewed as an entirely objective modeling method due to the effects of choosing different settings (Merow et al., 2014), we consider the final models not to be unnecessarily complex based on the knowledge of vegetation types, the environmental space, and the specific data set used in this study. In our study, the results may be influenced by several factors. Firstly, we compiled the presence‐only data from different sources, and it is highly likely that not all native occurrence records of the species have been included in this study. Secondly, the distributions of Dipterocarp trees are relatively well known across India, Bangladesh, Nepal, and Myanmar (e.g., Alam et al., 2008; Appanah & Turnbull, 1998; Champion & Seth, 1968; Chitale & Behera, 2012). This may be partly responsible for the higher number of species occurrence records in these areas compared to other native ranges. As the main objective of our study was to assess species vulnerability to climate change, we used only bioclimatic variables in the model. The realized climatic niche for the Dipterocarp trees that we describe here represents a close approximation to reality (Alam et al., 2008; Champion & Seth, 1968; Chitale & Behera, 2012). Future research needs to focus on mechanistic modeling of the Dipterocarp trees using detailed understanding of the physiological response of species to environmental factors (Pearson, 2010).

## Conflict of Interest

None declared.
