# Supplementary material for: The impact of climate change on the distribution of two threatened Dipterocarp trees
Source: Ecol Evol. 2017 Mar 5;7(7):2238–48. doi: 10.1002/ece3.2846 (PMC5383467; doi:10.1002/ece3.2846)

**SUPPORTING INFORMATION**

**Table S1** The natural distribution of the two Dipterocarp trees can be categorized in the following eco-regions of the South and Southeast Asia.

| Dipterocarp Species/Forests | Eco-regions/Climatic regions |
| --- | --- |
| *Shorea robusta* | - Upper Gangetic Plains Moist Deciduous Forests |
|  | - Chhota-Nagpur Dry Deciduous Forests |
|  | - Eastern Highlands Moist Deciduous Forests |
|  | - Lower Gangetic Plains Moist Deciduous Forests |
|  | - Northern Dry Deciduous Forests |
| *Dipterocarpus turbinatus* | - Brahmaputra Valley Semi-Evergreen Forests |
|  | - Cardamom Mountains Rain Forests |
|  | - Luang Prabang Montane Rain Forests |
|  | - Meghalaya Subtropical Forests |
|  | - Mizoram-Manipur-Kachin Rain Forests |
|  | - Northern Annamites Rain Forests |
|  | - Lower Gangetic Plains Moist Deciduous Forests |
|  | - Northern Khorat Plateau Moist Deciduous Forests |
|  | - Southern Annamites Montane Rain Forests |
|  | - Southeastern Indochina Dry Evergreen Forests |

**Table S2** The result of correlation test of the 19 environmental variables initially selected for the MaxEnt models.

| Variables | BIO1 | BIO2 | BIO3 | BIO4 | BIO5 | BIO6 | BIO7 | BIO8 | BIO9 | BIO10 | BIO11 | BIO12 | BIO13 | BIO14 | BIO15 | BIO16 | BIO17 | BIO18 | BIO19 |
| --- | --- | --- | --- | --- | --- | --- | --- | --- | --- | --- | --- | --- | --- | --- | --- | --- | --- | --- | --- |
| BIO1 | 1.00 | 0.05 | 0.33 | -0.43 | **0.86** | **0.90** | -0.20 | **0.92** | **0.93** | **0.93** | **0.95** | 0.09 | 0.14 | -0.16 | 0.35 | 0.13 | -0.21 | -0.17 | 0.01 |
| BIO2 | 0.05 | 1.00 | -0.37 | 0.58 | 0.46 | -0.32 | **0.83** | 0.18 | 0.06 | 0.29 | -0.15 | -0.60 | -0.41 | -0.44 | 0.61 | -0.44 | -0.48 | -0.43 | -0.29 |
| BIO3 | 0.33 | -0.37 | 1.00 | **-0.87** | -0.06 | 0.62 | **-0.79** | 0.14 | 0.38 | 0.03 | 0.57 | 0.31 | 0.16 | 0.22 | -0.38 | 0.16 | 0.19 | 0.04 | 0.29 |
| BIO4 | -0.43 | 0.58 | **-0.87** | 1.00 | 0.02 | **-0.74** | **0.90** | -0.19 | -0.43 | -0.09 | -0.68 | -0.47 | -0.31 | -0.15 | 0.39 | -0.33 | -0.13 | -0.13 | -0.21 |
| BIO5 | **0.86** | 0.46 | -0.06 | 0.02 | 1.00 | 0.59 | 0.30 | **0.84** | **0.82** | **0.97** | 0.69 | -0.24 | -0.08 | -0.32 | 0.62 | -0.10 | -0.38 | -0.43 | -0.11 |
| BIO6 | **0.90** | -0.32 | 0.62 | **-0.74** | 0.59 | 1.00 | -0.59 | **0.73** | **0.86** | **0.71** | **0.98** | 0.29 | 0.25 | 0.02 | 0.04 | 0.24 | -0.02 | -0.07 | 0.16 |
| BIO7 | -0.20 | **0.83** | **-0.79** | **0.90** | 0.30 | -0.59 | 1.00 | -0.02 | -0.20 | 0.14 | -0.47 | -0.58 | -0.37 | -0.35 | 0.58 | -0.39 | -0.36 | -0.34 | -0.29 |
| BIO8 | **0.92** | 0.18 | 0.14 | -0.19 | **0.84** | **0.73** | -0.02 | 1.00 | **0.81** | **0.92** | **0.80** | 0.04 | 0.10 | -0.19 | 0.45 | 0.10 | -0.22 | -0.07 | -0.07 |
| BIO9 | **0.93** | 0.06 | 0.38 | -0.43 | **0.82** | **0.86** | -0.20 | **0.81** | 1.00 | **0.87** | **0.90** | 0.05 | 0.11 | -0.14 | 0.35 | 0.09 | -0.19 | -0.28 | 0.07 |
| BIO10 | **0.93** | 0.29 | 0.03 | -0.09 | **0.97** | **0.71** | 0.14 | **0.92** | **0.87** | 1.00 | **0.79** | -0.11 | 0.02 | -0.24 | 0.56 | 0.00 | -0.30 | -0.29 | -0.06 |
| BIO11 | **0.95** | -0.15 | 0.57 | -0.68 | 0.69 | **0.98** | -0.46 | **0.80** | **0.90** | **0.79** | 1.00 | 0.23 | 0.21 | -0.08 | 0.16 | 0.21 | -0.13 | -0.12 | 0.09 |
| BIO12 | 0.09 | -0.60 | 0.31 | -0.47 | -0.24 | 0.29 | -0.58 | 0.04 | 0.05 | -0.11 | 0.23 | 1.00 | **0.90** | **0.71** | -0.21 | **0.94** | **0.75** | **0.71** | **0.73** |
| BIO13 | 0.14 | -0.41 | 0.16 | -0.31 | -0.08 | 0.25 | -0.37 | 0.10 | 0.11 | 0.02 | 0.21 | **0.90** | 1.00 | 0.11 | 0.13 | **0.98** | 0.14 | 0.55 | 0.35 |
| BIO14 | -0.16 | -0.44 | 0.22 | -0.15 | -0.32 | 0.02 | -0.35 | -0.19 | -0.14 | -0.24 | -0.08 | **0.71** | 0.11 | 1.00 | -0.49 | 0.14 | **0.94** | 0.29 | 0.30 |
| BIO15 | 0.35 | 0.61 | -0.38 | 0.39 | 0.62 | 0.04 | 0.58 | 0.45 | 0.35 | 0.56 | 0.16 | -0.21 | 0.13 | -0.49 | 1.00 | 0.07 | -0.52 | -0.28 | -0.13 |
| BIO16 | 0.13 | -0.44 | 0.16 | -0.33 | -0.10 | 0.24 | -0.39 | 0.10 | 0.09 | 0.00 | 0.21 | **0.94** | **0.98** | 0.14 | 0.07 | 1.00 | 0.18 | 0.61 | 0.30 |
| BIO17 | -0.21 | -0.48 | 0.19 | -0.13 | -0.38 | -0.02 | -0.36 | -0.22 | -0.19 | -0.30 | -0.13 | **0.75** | 0.14 | **0.94** | -0.52 | 0.18 | 1.00 | 0.36 | 0.31 |
| BIO18 | -0.17 | -0.43 | 0.04 | -0.13 | -0.43 | -0.07 | -0.34 | -0.07 | -0.28 | -0.29 | -0.12 | **0.71** | 0.55 | 0.29 | -0.28 | 0.61 | 0.36 | 1.00 | 0.00 |
| BIO19 | 0.01 | -0.29 | 0.29 | -0.21 | -0.11 | 0.16 | -0.29 | -0.07 | 0.07 | -0.06 | 0.09 | **0.73** | 0.35 | 0.30 | -0.13 | 0.30 | 0.31 | 0.00 | 1.00 |

**Table S3** The CMIP5 Global Climate Models used in the analysis:

| Model Name | Modelling Centre (or Group) | Institute ID |
| --- | --- | --- |
| ACCESS1.0 | Commonwealth Scientific and Industrial Research Organization (CSIRO) and Bureau of Meteorology (BOM), Australia | CSIRO-BOM |
| GFDL-CM3 | NOAA Geophysical Fluid Dynamics Laboratory | NOAA GFDL |
| HadGEM2-ES | Met Office Hadley Centre (contributed by Instituto Nacional de Pesquisas Espaciais) | MOHC (INPE) |

**Figure S1.** The bias layer created for the (a) Sal (*Shorea robusta*) and (b) Garjan (*Dipterocarpus turbinatus*) species to limit the background points to the occurrence areas for the species.

**Figure S2.** The jackknife test results for environmental variables: (a) *Shorea robusta* model, and (b) *Dipterocarpus turbinatus* model. The graph depicts the training gain of each variable if the model was run in isolation, and compares it to the training gain with all the variables. Annual precipitation (BIO12) was the most significant variable with highest gain when used in isolation for both models.


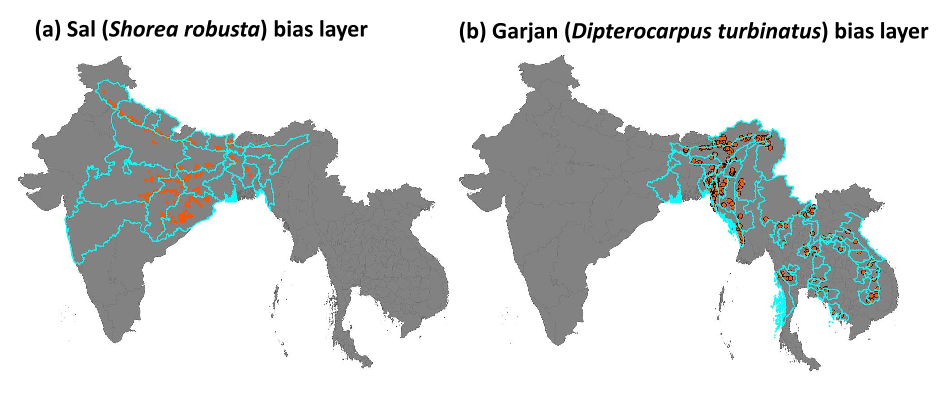


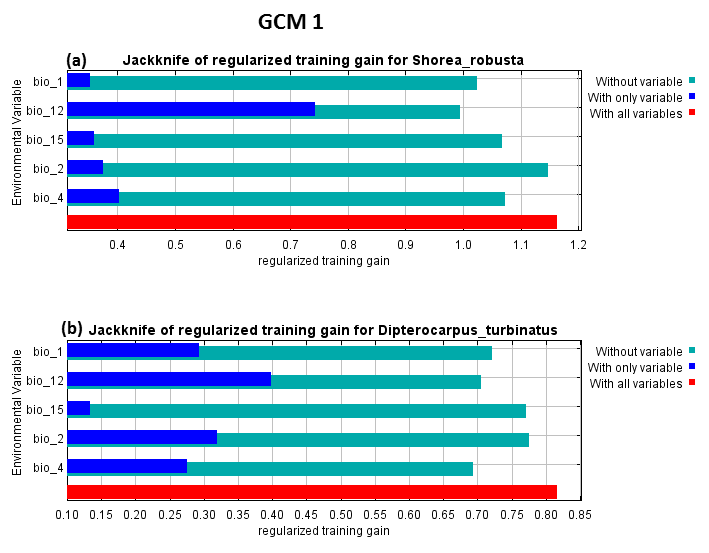

Supplement: Supplementary file 1 [file ECE3-7-2238-s001.docx]
